# Protein-caged zinc porphyrin as a carbonic anhydrase mimic for carbon dioxide capture

**DOI:** 10.1038/s41598-020-76482-8

**Published:** 2020-11-11

**Authors:** Haixia Chi, Han Chen, Kai Gong, Xiaoqiang Wang, Youming Zhang

**Affiliations:** 1 Shandong University-Helmholtz Institute of Biotechnology, State Key Laboratory of Microbial Technology, Shandong University, Qingdao, 266237 China; 2 State Key Laboratory of Heavy Oil Processing, College of Chemical Engineering, China University of Petroleum (East China), Qingdao, 266580 China

**Keywords:** Biophysics, Biotechnology, Materials science, Nanoscience and technology

## Abstract

Zinc tetraphenylporphyrin (Zn-TPP) solubilized by GroEL protein cage was prepared as a supramolecular mimic of carbonic anhydrase (CA) for CO_2_ capture. It is shown that the soluble Zn-TPP-GroEL complex can be formed easily by detergent dialysis. The Zn-TPP/GroEL binding ratio was found to increase with their dialysis ratio until reaching the maximum of about 30 porphyrins per protein cage. Moreover, the complex showed hydrase activity that catalyzes the CO_2_ hydration in HCO_3_^−^ and H^+^. It is further seen that the catalytic activity of Zn-TPP-GroEL was about one-half of that of a bovine CA at 25 °C. On the other hand, as the temperature was increased to 60 °C close to an industrial CO_2_ absorption temperature, the natural enzyme lost function while Zn-TPP-GroEL exhibited better catalytic performance indicative of a higher thermal stability. Finally, we demonstrate that the GroEL-solubilized Zn-TPP is able to accelerate the precipitation of CO_2_ in the form of CaCO_3_ and has better long-term performance than the bovine CA. Thus a new type of nano-caged system mimicking natural CAs for potential applications in carbon capture has been established.

## Introduction

Carbon dioxide (CO_2_) is a major contributor to global warming, and the burning of fossil fuels is the primary source of CO_2_ emissions^1–3^. At present considerable effort is being expanded to develop a variety of cost-effective CO_2_ capture technologies capable of curbing the emissions of this heat-trapping gas. One possibility rests on the use of naturally evolved carbonic anhydrases (CAs) and their mimics. CAs are a superfamily of zinc metalloenzymes that have been long known to catalyze the reversible hydration of CO_2_ in bicarbonate ions and protons in vivo^4–6^. Though this reaction may occur spontaneously, it is too slow to meet the needs of life. Thanks to the assistance of CA enzymes with a turnover number *k*_cat_ of > 10^6^ s^−1^, the reaction is made possible within biologically relevant timescales^7^.

On the other hand, the CO_2_ hydration is still considered as a rate-limiting step in our endeavor to control carbon emissions^8–10^. Inspired by the in vivo enzymatic reaction, CO_2_ sequestration with CAs was widely explored for promoting the hydration reaction^11–15^. While considerable progress has been made in this aspect, the disadvantages of CAs themselves might restrict their broad industrial applications. For instance, one major concern is their low operational stability despite unparalleled catalytic activity^16,17^. This issue is especially important given the harsh conditions found in CO_2_ capture, like high-temperature flue gas (40–60 °C or even higher).

The strategy of constructing CA mimics is of particular interest among those developed to conquer potential limitations of CAs^18–20^. Up to date a majority of mimics investigated are structural analogs of the CA active sites that mostly comprise a zinc atom coordinated by N atoms provided by the imidazoleside chains of vicinal His residues. As an example, the so-called zinc cyclen is one of most efficient mimics ever identified, which consists of a zinc atom coordinated to four N atoms in a cyclic amine ligand^21^. Notably, it exhibited excellent CO_2_ hydration activity even under rigorous conditions resembling those in an industrial carbon capture process^22^. Thus the employment of CA mimics represents an interesting possibility.

**Figure 1:**
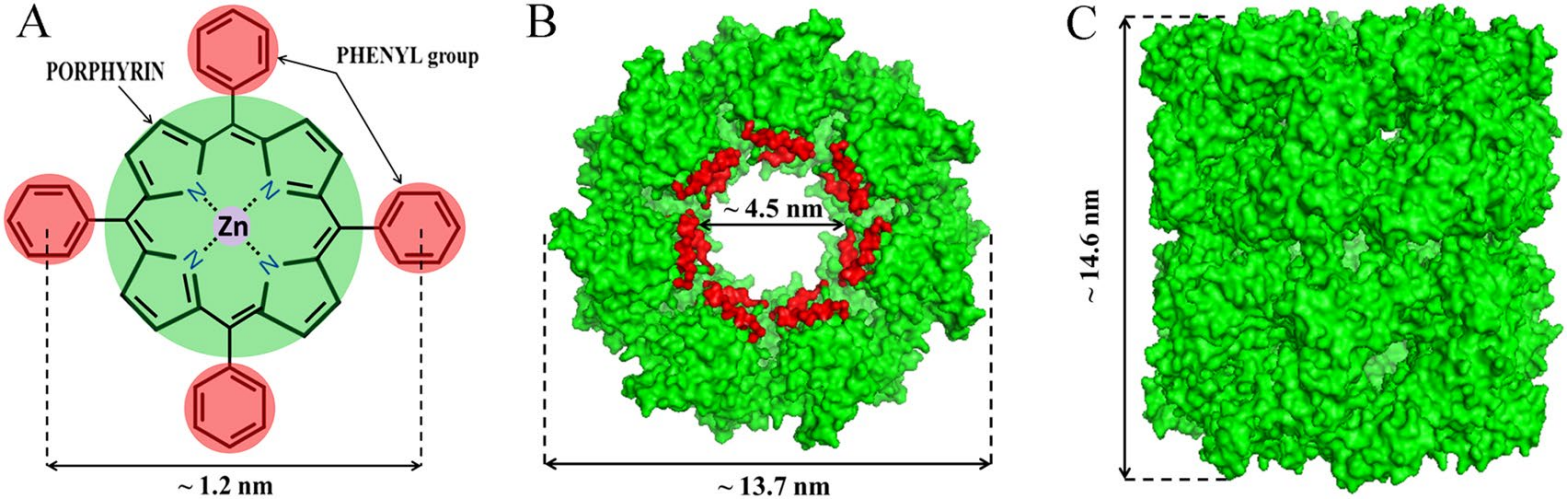
Structures of Zn-TPP and GroEL studied in this work. (**A**) Chemical structure of Zn-TPP, in which four phenyl groups (red) are attached to the central porphyrin ring (green), and the central atom is zinc (purple). (**B**) End-view of GroEL showing the hydrophobic lining (red) facing its central cavity. (**C**) Side-view of the GroEL protein cage. Pymol was used to generate GroEL structures (PDB code 1SS8). Zn-TPP and GroEL are rendered at different scales.

Here we wish to present preliminary results using commercially available zinc tetraphenylporphyrin (Zn-TPP) as a CA mimic, taking advantage of the natural coordination between the central Zn atom and four N atoms from the porphyrin (Fig. 1A), similar to CA active sites and the aforementioned zinc cyclen as well. The four phenyl groups attached to the porphyrin, however, render high hydrophobicity to Zn-TPP. To address this, the hydrophobic central cavity of GroEL protein cage from *E. coli* was harnessed to assemble Zn-TPP molecules to form a water-soluble, supramolecular nanostructure with CA-like activity. The crystal structure of GroEL is as shown in Fig. 1B,C,which is composed of two stacked seven-membered rings, creating two disconnected hydrophobic cavities with a diameter and depth of ~ 4.5 nm each^23^. It is worth mentioning that GroEL is highly resistant against thermal or chemical denaturation, which for example becomes denatured only at a temperature of up to 70 °C or in the presence of more than 3.2 M urea^24,25^. Moreover, we envisioned that the CO_2_ hydration takes place in GroEL cavity with hydrophobic lining, better simulating the hydrophobic pocket required for CO_2_ positioning in natural CAs than purely soluble CA mimics.

The soluble Zn-TPP-GroEL complex was prepared by detergent dialysis, followed by a systematic characterization on its nano-structure, catalytic activity, stability and performance in CO_2_ precipitation. This is the first study of zinc porphyrin or its supramolecular assembly with a protein cage that is employed as a CA mimic. The easy availability of Zn-TPP as well as recombinant GroEL proteins^26^, along with the dialysis method, also renders the CA mimic preparation cost-effective.

## Materials and methods

### Materials

Zinc tetraphenylporphyrin, Bromothymol blue (BTB) and carbonic anhydrase from bovine erythrocytes were purchased from Sigma-Aldrich (Catalog# 252174; 18470; C2624). The *E. coli* strain BL21 (DE3) was used for GroEL overexpression. The protein was then purified to ~ 95% purity by anion-exchange chromatography and gel filtration, as previously reported^26^.

### Construction and characterization of Zn-TPP-GroEL supramolecular assembly

Zn-TPP-GroEL was prepared as follows. The hydrophobic Zn-TPP pre-dissolved in N, N-dimethylformamide was diluted with Triton-X-100 aqueous solution to a concentration of 1 mM. The Triton-X-100 solution with a micelle concentration of 3 mM was made in 12 mM phosphate buffered saline (PBS), pH 7.0. Next, the Triton-X-100-solubilized Zn-TPP was further diluted with PBS to 20 μM, followed by mixing with the same volume of GroEL solution (2 μM in 12 mM PBS, pH 7.0). The mixture was then placed in a dialysis bag, and dialyzed against PBS for 3 days at 4 °C, during which PBS was replaced every 8 h. After 3 days, the dialyzed sample was centrifuged at 13,000 g for 10 min, and the supernatant was collected for subsequent characterizations. This usually produces a Zn-TPP-GroEL conjugate with a Zn-TPP/GroEL ratio of 10:1, as determined by UV–vis spectroscopy to quantify Zn-TPP. Preliminary Bradford protein assay suggested no detectable protein loss during detergent dialysis. Zn-TPP-GroEL assemblies with different Zn-TPP/GroEL ratios were prepared in the same manner.

Transmissionelectron microscope (TEM) images were captured on a JEOL JEM-1400Plus instrument at 120 kV acceleration voltage to study the morphology and architecture of Zn-TPP-GroEL and GroEL. Shimadzu UV-2450 spectrophotometer was used to record the UV–vis absorption spectrum of Zn-TPP-GroEL. The amount of Zn-TPP entrapped in GroEL cage was calculated with Lambert–Beer law using an extinction coefficient of 5.74 × 10^5^ M^−1^·cm^−1^ at 422 nm. This combined with the GroEL amount determined by Bradford protein assay was used to estimate the binding ratio of porphyrins in the protein cage. Fluorescence spectra were measured on a Horiba Jobin Yvon Fluoromax 4 spectrofluorometer, with an excitation wavelength of 424 nm and a slit width of 4 nm^27^.

### Hydrase activity of Zn-TPP-GroEL

Wilbur-Anderson method was employed to determine the hydrase activity of Zn-TPP-GroEL, as described previously^28^. Briefly, 100 μL of 0.05% BTB was mixed with 1 mL of pre-chilled assay buffer (50 mM Tris–HCl, 100 mM sodium sulfate, pH 8.0). To this mixture Zn-TPP-GroEL was then added to a final concentration of 0.1 μM (as Zn-TPP). The reaction was initiated by adding 500 μL of pre-chilled CO_2_-saturated water, and the changes of pH and color of the reaction system were monitored. A pH electrode inserted into the assay solution was used to measure the pH change. The color change was recorded with a camera. A bovine CA or GroEL in place of the Zn-TPP-GroEL was used as a positive control or a negative control (blank). The hydrase activity was expressed as Wilbur-Anderson unit (WAU), which was determined by the formula of (T_0_ − T)/T. T_0_ (blank reaction) and T (catalyzed reaction) were recorded as the time (in seconds) taken for pH dropping from 8.0 to 7.0 in negative control and in the presence of catalytic species, respectively.

### Catalytic kinetics of Zn-TPP-GroEL

The ability of Zn-TPP-GroEL to capture CO_2_ was evaluated as previously reported with minor modifications^28^. First, Zn-TPP-GroEL assay solution (0.1 μM as Zn-TPP) was prepared using 100 mM HEPES buffer (pH 8.0). CO_2_ was then blown into this solution at a constant rate of 100 mL/min. Meanwhile, a pH electrode was inserted into the solution to detect pH change until it reached a constant level. The pH value recorded every 15 s during CO_2_ blowing was plotted as a function of time. The rate of pH decrease was used to estimate the catalytic kinetics of Zn-TPP-GroEL, which was also compared to that with a bovine CA (0.1 μM) or only GroEL in place of the Zn-TPP-GroEL. The reaction was tested at two temperatures: 25 °C and 60 °C.

### Catalytic CO_2_ precipitation

The effect of Zn-TPP-GroEL on CO_2_ precipitation to form CaCO_3_ was investigated by directly mixing 1 mL of Zn-TPP-GroEL solution (0.1 μM as Zn-TPP), 0.06 g of CaCl_2_·2H_2_O and 1 mL of Tris buffer solution containing 0.168 g Tris. To this mixture 4 mL of CO_2_-saturated water was added to initiate the precipitation reaction, which was then kept at 25 °C for 2 h with slow orbital shaking, followed by filtration and drying. Next, the precipitate was weighed to quantify the CaCO_3_ produced in the presence of Zn-TPP-GroEL, and compared to the case with a bovine CA (positive control) or the empty GroEL (negative control). To test the long-term performance of Zn-TPP-GroEL, we incubated the complex with varied binding ratios at 25 °C for up to 4 weeks and then measured their effect on CO_2_ precipitation under the aforementioned conditions after a brief centrifugation.

## Results and discussion

### Preparation of Zn-TPP-GroEL complex

**Figure 2:**
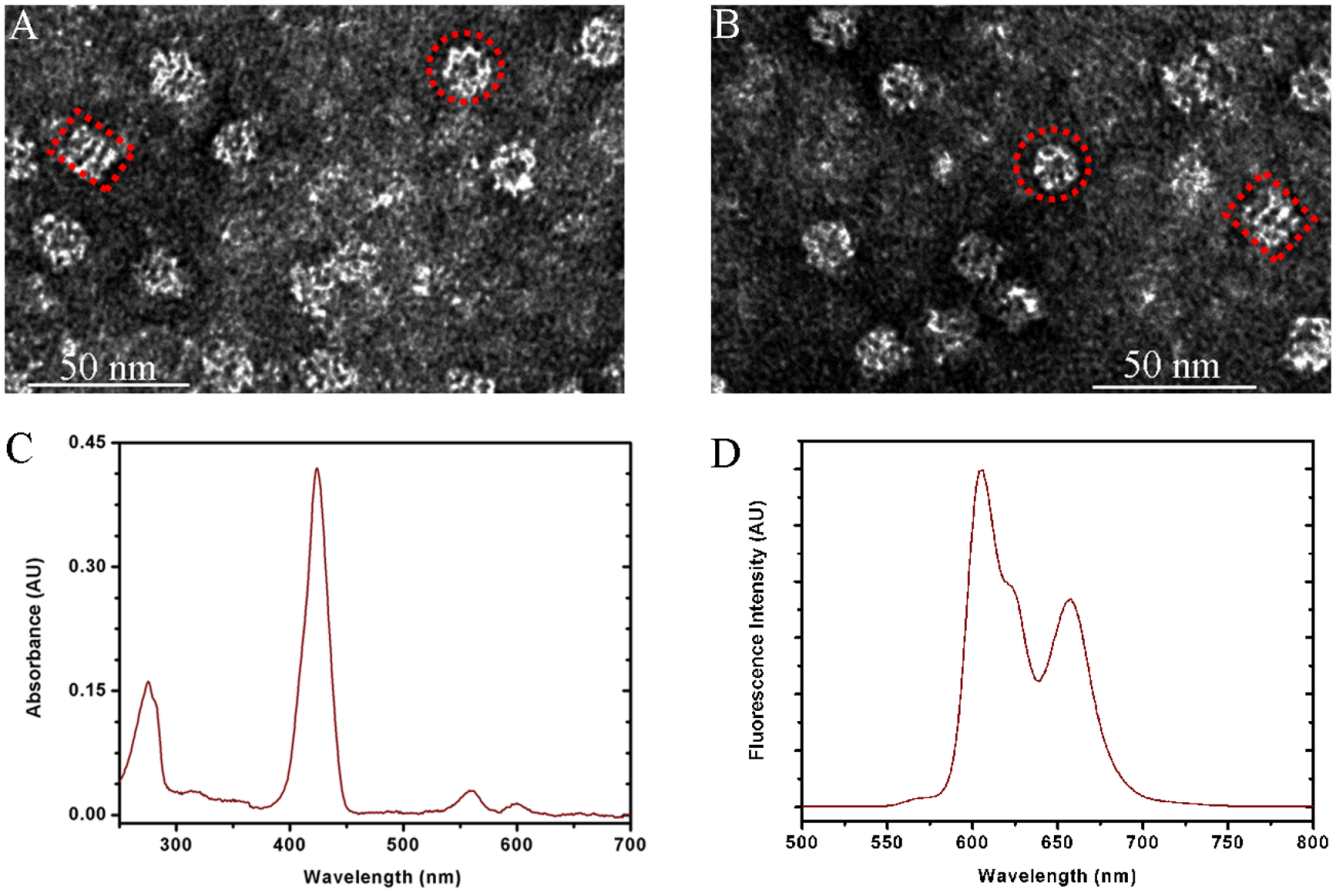
Characterization of the Zn-TPP-GroEL complex. (**A**) TEM micrograph of Zn-TPP-GroEL compared to (**B**) that of GroEL alone. As an example, the end-view or side-view of GroEL is marked with red circle or rectangle. (**C**) UV–vis spectrum of Zn-TPP-GroEL. (**D**) Fluorescence spectrum of Zn-TPP-GroEL (λ_ex_ = 424 nm).

The central cavity of GroEL is lined with hydrophobic amino acid residues evolutionarily optimized for capturing non-native substrate proteins with exposed hydrophobic surfaces for assisted folding^23^. This is distinct from other natural protein cages, and has been exploited as a smart carrier for loading and delivering hydrophobic drugs, nanoparticles or membrane proteins^29–31^. Herein, soluble complexes of GroEL with Zn-TPP were constructed as a CA mimic by dialysis to remove the detergent used to pre-disperse Zn-TPP in aqueous solution. Figure 2A shows the TEM micrograph of the as-prepared Zn-TPP-GroEL, in which the end-view and side-view of GroEL are clearly visible (red marks as an example). Notably, despite its high hydrophobicity, Zn-TPP binding to GroEL does not disrupt the integrity and morphology of the protein cage, as Zn-TPP-GroEL and GroEL are identical in size and shape (Fig. 2A,B). On the other hand, the UV–vis analysis indicates the presence of both porphyrin and protein in the Zn-TPP-GroEL. As can be seen from Fig. 2C, the absorption peaks at 424 nm, 560 nm and 600 nm could be ascribed to the Soret band and two Q bands of Zn-TPP^32^, while the absorption peak at 276 nm is presumably due to the absorbance of GroEL protein cage. Moreover, the fluorescence emission spectrum of Zn-TPP-GroEL reveals two primary emission bands at 605 nm and 658 nm (Fig. 2D), in agreement with those reported previously with Zn-TPP^33^.

The above data can be interpreted in support of an efficient transfer of Zn-TPP from a detergent-solubilized state to a detergent-free soluble complex with GroEL as Zn-TPP itself is unstable in an aqueous solution without the aid of the protein cage, although it is tricky at the current stage to observe or verify directly the presence of Zn-TPP inside GroEL protein cage. From a thermodynamic standpoint, it is conceivable that GroEL hydrophobic cavity presents a beneficial nanocompartment that would probably shield Zn-TPP from the aqueous solution after removal of detergent by dialysis. Similar method has been used for generating membrane protein-GroEL complex, with the capacity of solubilization estimated to be two molecules (or 52 kDa) per GroEL cage^31^. By increasing the amount of Zn-TPP in the dialysis mixture and measuring the soluble component after dialysis, the Zn-TPP/GroEL binding ratio was found to increase accordingly, with the maximum ratio determined to be around 30:1 by UV–vis spectroscopy, which is equal to ~ 20 kDa Zn-TPP loading on a mass basis. Since Zn-TPP is significantly smaller than a membrane protein, it is reasonable that much more Zn-TPP molecules can be packed in GroEL chamber. However, the maximum encapsulation mass of the porphyrin in the protein cage is still much less than the membrane protein, possibly due to the steric hindrance among the resident Zn-TPP molecules in GroEL cavity.

### The hydrase activity of Zn-TPP-GroEL

**Figure 3:**
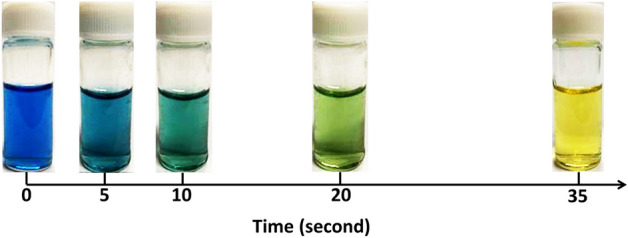
Wilbur-Anderson method was used for colorimetric identification of hydrase activity of Zn-TPP-GroEL. The reaction mixture containing Zn-TPP-GroEL and BTB dye showed a quick color change from blue to yellow.

We next studied the hydrase activity of Zn-TPP-GroEL using a colorimetric method developed by Wilbur and Anderson^34^, which introduces cold CO_2_-saturated water into the assay solution containing BTB as a pH indicator. Figure 3 demonstrates the color change of Zn-TPP-GroEL solution with this treatment. The solution was observed to shift from blue to green and then to yellow within 35 s, corresponding to a pH change from basic to neutral and then to acidic condition^28^. This is due to CO_2_ hydration and then dissociation to HCO_3_^−^ and H^+^, thus leading to a decrease of pH of the solution. A similar trend was also seen for the control without Zn-TPP-GroEL, but it took more than 9 min to achieve the same level of color conversion (data not shown). Thus Zn-TPP-GroEL exhibited the hydrase activity to accelerate the hydration of CO_2_ and its hydrolysis.

**Figure 4:**
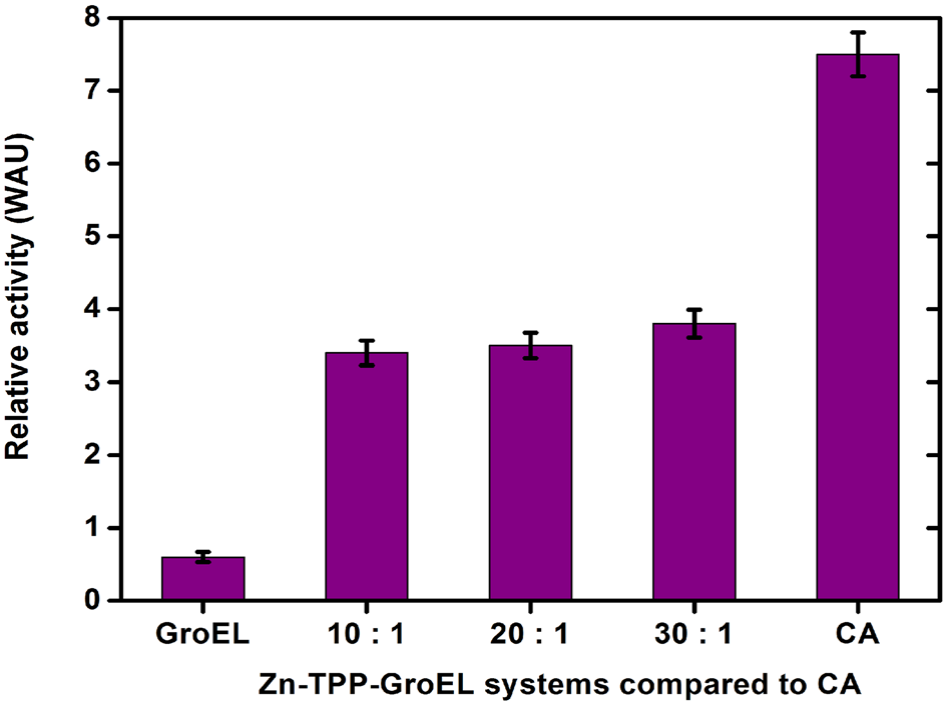
Hydrase activities of the Zn-TPP-GroEL complexes with different porphyrin/protein binding ratios (10:1, 20:1 and 30:1) at 25 °C, compared with CA (positive control) and GroEL alone (negative control). The hydrase activity was calculated as the difference in the initial rate of CO_2_ hydration between the blank and the samples. The activity value represents the average of triplicate measurements. The vertical bar shows the standard deviation.

The hydrase activity of Zn-TPP-GroEL was quantified in terms of Wilbur-Anderson units (WAU). As shown in Fig. 4, the WAU for Zn-TPP-GroEL was 3.8 at the maximum porphyrin/protein binding ratio (30:1), and decreased somewhat at lower binding ratios (*e.g.*, 20:1 and 10:1). By comparison, the WAU for a bovine CA was almost twice as high as Zn-TPP-GroEL (30:1). Although the concentration of Zn-TPP entrapped in GroEL (30:1) are the same as the CA with one catalytically active site, proper secondary coordination environment or other interactions as found in the natural enzymes are likely required for more efficient catalytic hydration of CO_2_^35^. On the other hand, GroEL alone gave a very low WAU, mirroring a negligible ability of the protein cage itself to promote the hydration process. Additionally, note that the increase of Zn-TPP-GroEL activity is seemingly out of proportion to the increase of porphyrin/protein binding ratio. This might be due to the increase of Zn-TPP packing density in GroEL chamber, which limits the mass transfer process from the active sites to the open mouth of the protein cage or its porous wall^23^.

**Figure 5:**
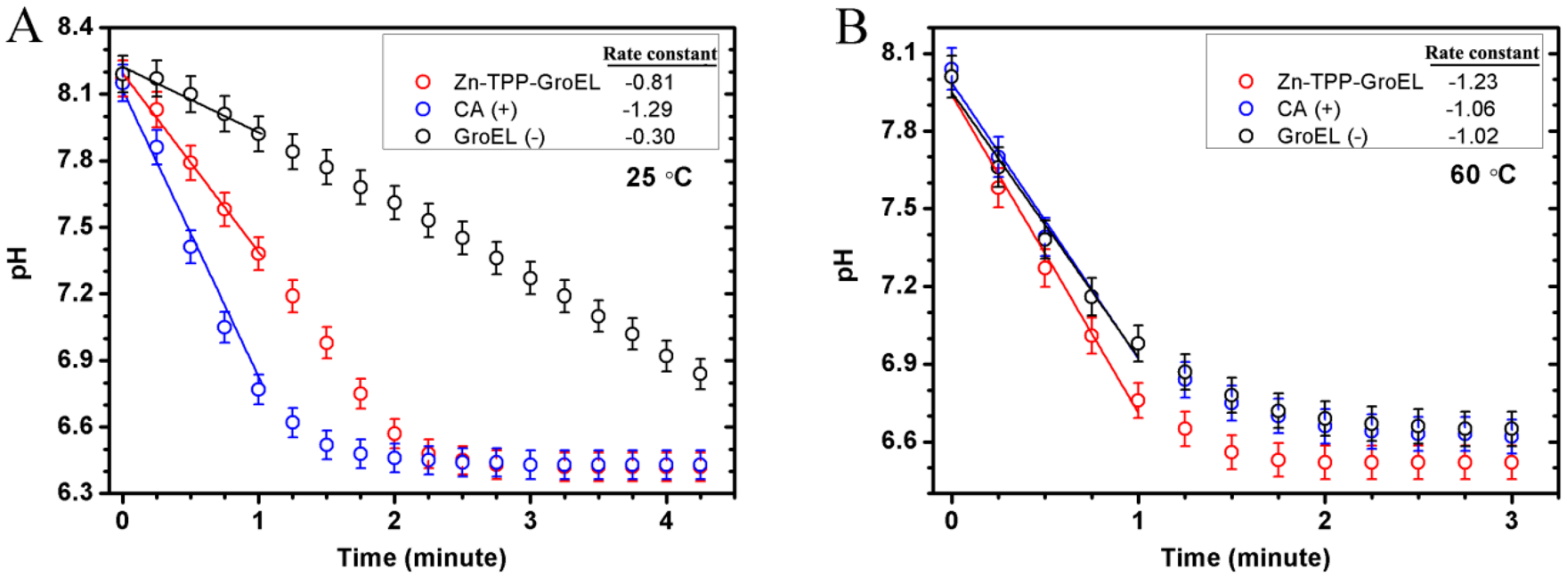
The catalytic kinetics of Zn-TPP evaluated by measuring the pH reduction over time, compared with CA (positive control) and GroEL alone (negative control). Measurements were performed at both 25 °C (**A**) and 60 °C (**B**). The reaction rate constants within the first minute were used to compare the catalytic activity. The pH values shown are the means of triplicate measurements.

We next investigated the catalytic kinetics of Zn-TPP-GroEL by blowing CO_2_ into the assay solution and monitoring the pH change over time. Figure 5A shows the results obtained at 25 °C, in which the initial reductions of pH all follow a pseudo-first order kinetics, yielding a reaction rate constant of − 0.81 min^−1^ for Zn-TPP-GroEL, and − 1.29 min^-1^ or − 0.30 min^−1^ for CA (positive control) or GroEL alone (negative control) (insert of Fig. 5A). The turn-off numbers for the CA mimic and natural enzyme were − 8.10 × 10^6^ L·mol^−1^·min^−1^ and − 1.29 × 10^7^ L·mol^−1^·min^−1^. All the pH reductions corresponding to the increase of H^+^ concentration could be attributed to CO_2_ hydration and then hydrolysis to form H^+^, either in a spontaneous or catalyzed manner. While Zn-TPP-GroEL significantly accelerated the CO_2_ hydration and hence pH reduction compared with GroEL, it was less efficient than CA, according to the reaction rate constants. However, when the temperature was increased to 60 °C, Zn-TPP-GroEL exhibited better catalytic activity, giving a reaction rate constant of − 1.23 min^−1^ and turn-off number of − 1.23 × 10^7^ L·mol^−1^·min^−1^, which are greater in absolute value than those for CA and empty GroEL (Fig. 5B). Moreover, the kinetic curve for the natural enzyme almost overlapped with that for the protein cage alone, indicating a nearly complete inactivation of CA, probably due to structure denaturation at this temperature^22^. By contrast, that GroEL becomes denatured only at a temperature up to 70 °C is believed to impart high thermostability to Zn-TPP-GroEL^24^, which might also account for its good catalytic activity even at 60 °C. Combined, Zn-TPP-GroEL had a lower catalytic activity than CA at room temperature, whereas it exhibited better catalytic performance and stability at high temperatures detrimental to most enzymes, making it a robust material with great potential for industrial carbon capture operated usually at 40–60°C^22^.

#### Zn-TPP-GroEL for CO_2_ precipitation

**Figure 6:**
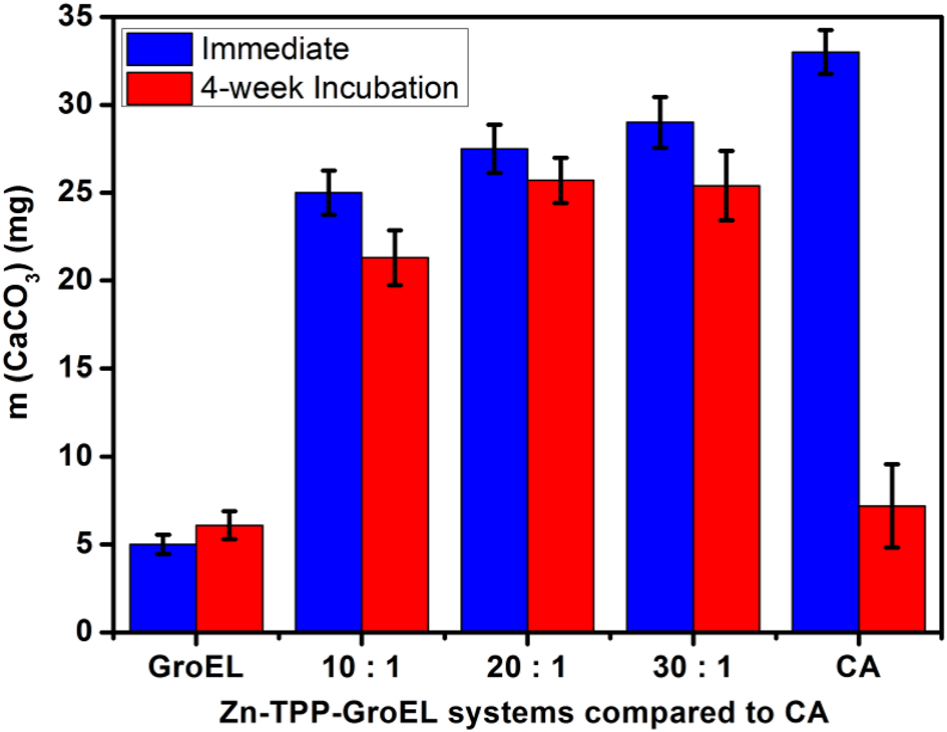
CaCO_3_ precipitation mediated by Zn-TPP-GroEL with different porphyrin/protein binding ratios at 25 °C, compared with CA (positive control) and the empty GroEL cage (negative control). Zn-TPP-GroEL was tested right after its preparation (blue bars) or after 4-week incubation at 25 °C (red bars). The precipitate amount represents the average of triplicate measurements. The vertical bar shows the standard deviation.

The feasibility of Zn-TPP-GroEL for catalyzing CO_2_ precipitation in the form of CaCO_3_ was also probed as previously reported with minor modifications^36^. Zn-TPP-GroEL systems either freshly prepared or incubated at 25 °C for 4 weeks were tested in this reaction. The results for the freshly prepared systems demonstrated that the precipitation was dependent on the porphyrin/protein binding ratio in Zn-TPP-GroEL to some extent (blue bars in Fig. 6). As the binding ratio increased from 10:1 to 30:1, the amount of CaCO_3_ obtained within 2 h also increased from 25.2 mg to 28.9 mg. With natural CA or GroEL at a concentration equal to the porphyrin or protein cage in Zn-TPP-GroEL (30:1), the weight of CaCO_3_ was 32.9 mg and 4.8 mg, respectively. It can be seen that Zn-TPP-GroEL exhibited a notable ability to promote the CO_2_ precipitation to form CaCO_3_ when compared with GroEL, underling the important role of Zn-TPP as a mimic of CA active site in this process. Again, CA demonstrated an even higher catalytic activity than Zn-TPP-GroEL in CaCO_3_ formation, in agreement with the above kinetic results. To our best knowledge, no CA mimics with an activity better than natural CAs at lower temperatures (*e.g.*, ≤ 37 °C) have been reported yet.

On the other hand, when Zn-TPP-GroEL was pre-incubated at 25° C for 4 weeks, the catalyzed CO_2_ precipitation yielded less CaCO_3_ than its freshly prepared counterpart, suggesting a decrease of catalytic performance (red bars in Fig. 6). Notably, CA almost lost its catalytic activity after 4-week incubation with the amount of collected CaCO_3_ close to that obtained in the negative control with empty GroEL cage. Thus Zn-TPP-GroEL exhibited better long-term catalytic performance than the natural enzyme under the tested conditions. Meanwhile, in the hydration process, no side-reactions or poisonous species that may affect catalytic activity are anticipated to form. Also, GroEL cage itself has good thermal stability under the tested conditions. Thus, the biomimetic catalyst should be used again with the initial performance, especially when combined with a suitable immobilization technique. Activity and stability are two important aspects for evaluating the catalytic performance of catalytic materials and should be balanced in practical applications.

## Conclusions

We have constructed a new type of supramolecular CA mimic with zinc porphyrin and GroEL protein cage based on non-specific hydrophobic interactions. It is shown that soluble conjugates of GroEL with porphyrins can be constructed efficiently by detergent dialysis. A maximum binding ratio of about 30 porphyrins per GroEL cage was achieved. The protein-caged porphyrin exhibited the hydrase activity catalyzing the hydration of CO_2_ and its hydrolysis. As compared with a natural CA, the porphyrin-GroEL system showed a lower catalytic activity at room temperature. However, when the temperature was increased to 60 °C that led to the inactivation of the natural enzyme, our system exhibited better catalytic performance and stability. Additionally, we demonstrate that protein-caged porphyrin is able to catalyze the CO_2_ precipitation to form CaCO_3_ with better long-term performance than the CA. The porphyrin-GroEL system we designed to simulate natural CAs may be used under expected industrial conditions, such as CO_2_ precipitation from industrial flue gases with high temperature.
